# Influence of an Educational Intervention on Eating Habits in School-Aged Children

**DOI:** 10.3390/children9040574

**Published:** 2022-04-17

**Authors:** María José Menor-Rodriguez, Jonathan Cortés-Martín, Raquel Rodríguez-Blanque, María Isabel Tovar-Gálvez, María José Aguilar-Cordero, Juan Carlos Sánchez-García

**Affiliations:** 1 Research Group CTS367, E.U.E. Campus Ourense, University of Vigo, 32616 Ourense, Spain; maria.jose.menor.rodriguez@sergas.es; 2 Research Group CTS1068, School of Nursing, Faculty of Health Sciences, University of Granada, 18071 Granada, Spain; jonathan.cortes.martin@gmail.com (J.C.-M.); jsangar@ugr.es (J.C.S.-G.); 3 Research Group CTS1068, School of Nursing Ceuta Campus, Faculty of Health Sciences, University of Granada, 51001 Ceuta, Spain; matoga@ugr.es; 4 Research Group CTS367, School of Nursing, Faculty of Health Sciences, University of Granada, 18071 Granada, Spain; mariajaguilar@telefonica.net

**Keywords:** health behavior, students, children, nutrition, prevention

## Abstract

A health promotion intervention directed at preventing unhealthy habits in relation to physical exercise, hygiene habits, diet and personal relationships can lead to a decrease in diseases during adulthood and obtaining a better quality of life. The intervention had a participative and multidisciplinary nature, and it was developed by health professionals, teachers and parents for school children. It promoted healthy lifestyle habits around food by working on four areas through video lessons, interactive talks, practical and theoretical–practical classes and an individual project on behalf of the student where they had to design healthy menus. We randomly selected, by stratified multistage sampling, three public primary education schools. A sample of 479 students registered during the 2014/2015 academic year aged between 6–12 years were included in the study. After the educational intervention, we found a significant reduction in the BMI of the students (*p* < 0.001). For breakfast, the overweight or obese children modified their usual food consumption after the intervention for a healthier breakfast based on fruit juice and bread instead of sugary snacks (*p* < 0.001). Regarding eating habits during the mid-morning break, there was a decrease in the consumption of sugary snacks (*p* < 0.001) and an increase in the consumption of fruits and sandwiches. We found that, after the intervention, the excessive consumption of pasta for the main meal reduced in favor of an increase in the consumption of proteins and different beans and pulses (*p* > 0.001). Educational interventions on healthy lifestyle habits carried out in children during their early years improve and correct unhealthy habits.

## 1. Introduction

The primary school stage constitutes a stable period of growth and of physical and intellectual development. A healthy diet is fundamental to a correct nutritional education because habits established at this early stage are difficult to change in adulthood [1,2,3]. The school years represent the crucial moment for the consolidation of healthy eating habits and physical exercise.

Health promotion is a strategy for the acquisition and development of personal skills related to health-related behavioral changes and the promotion of healthy lifestyles [4,5].

In Spain, the National Public Health Survey (ENSE 2012) [6] analyzes the population’s physical activity by age group and sex, providing healthcare information on 21508 Spanish homes. It is a periodic five-year research plan that retrieves information on different aspects of community health. This study showed that 12.1% of the population aged 5–14 years did not perform any physical exercise in their free time, and a sedentary lifestyle was twice as prevalent in girls (16.3%) than in boys (8.2%). In the year 2000, the enKid study [7] had already shown that the prevalence of overweight was at 12.4% and obesity at 13.9%.

The ALADINO [8] study is an epidemiological project on childhood obesity in Spain under the COSI [9] initiative that the OMS European Office has been developing since 2007 and to which 46 countries are currently subscribed. In 2011, the Aladino study [10], carried out in school children aged 6–10 years, revealed that 44.5% of school children had excess weight. It established the prevalence of overweight (≥p85 BMI) at 26.2% (26.7% male; 25.7% female) and of obesity at 18.3% (20.9% male; 15.5% female). These results observe a downward trend of obesity in successive years, remaining stable as of 2015 [11]. Currently, the most recent ALADINO study is from 2019 [12], where a cross-sectional descriptive study of anthropometric measurements obtained by direct measurement and associated factors obtained through questionnaires is carried out in a random sample of 16665 schoolchildren between 6–9 years of age in 276 primary education centers, obtaining, as the main result, that the prevalence of overweight and obesity in this type of schoolchildren in Spain continues to be high.

In the Galician Autonomous Community (Spain), the “Plan Xermola” [13] that studied the tendency of overweight and obesity compared the results obtained in the Aladino study and the enKid study and found a stabilization of the tendency of overweight, although an increase in obesity in girls was found. After analyzing the data, the prevalence of overweight was estimated at 25–30% and at 12–16% for obesity.

In several studies carried out in Spain, epidemiological evidence has identified overweight and obesity as risk factors for the development of chronic diseases [14,15] among the young, such as high blood pressure or type 2 diabetes [16,17]. The introduction of educational programs and nutritional interventions in our education system is essential in order to reduce the burden of this situation [18]. Such programs must be closely monitored in order to adjust them as much as possible to the continuously changing eating habits [19,20,21,22].

In 2005, the Spanish Ministry of Health and Consumers Affairs established the “Estrategia NAOS” [19], a platform that would include and promote all those initiatives that contribute to achieving the needed social change by the promotion of a healthy diet and preventing physical inactivity based on a series of specific challenges in different application areas. The development of these activities and the inclusion of new ones, along with the evaluation and surveillance of each one, will be key in maintaining a high rate of efficacy in the prevention of obesity [23].

Our hypothesis was that an educational intervention on healthy lifestyle habits in school-aged children will allow them to disregard their unhealthy habits regarding their diets [24,25,26]. Children will learn to eat properly, develop healthy eating habits and keep an active lifestyle so that when they grow up it will be less probable that they will develop chronic diseases such as obesity, cardiovascular diseases, non-insulin dependent diabetes, certain types of cancer, etc. [19]. They will also be more likely, as adults, to keep up these good lifestyle habits, to have a healthy diet and to continue to perform regular physical activity [27].

The aim of this study was to evaluate the influence of an educational intervention in the modification of nutritional habits and healthy diet in school-aged children.

This intervention aimed to promote healthy lifestyles, and its implementation in the school context should be seen as an improvement, since it will have an impact on children and their communities, contributing to an increased involvement and self-responsibility in the acquisition of knowledge, participation and decision making related to their eating habits.

## 2. Materials and Methods

This study achieved conformity with the CONSORT standards published in 2010 (“CONSORT Checklist”, 2017). It was approved by the Research Ethics Committee for the province of Granada and assigned File No. 13-06 and registered with the registration code 2014/130 in the CEI of Pontevedra-Vigo-Ourense (https://acis.sergas.gal/cartafol/Axentes-de-investigacion, accessed on 10 February 2014). At all times, the study was conducted in accordance with the provisions of the Declaration of Helsinki, as amended at the 64th WMA General Assembly, Fortaleza, Brazil, October 2013.

Informed consent was obtained from all subjects involved in the study.

The study design was an educational intervention with a semi-experimental design where we made measurements pre and post implementing the training in healthy lifestyle habits. The behavior model used is participant modeling, in which, the action was performed simultaneously with the child, who learned it by collaborating. Three public primary schools in the Galician Autonomous Community were randomly selected by stratified multistage sampling. The schools selected were CEIP Seixalbo school, CEIP Padre Crespo Xunqueira (both in the province of Ourense) and CEIP Igrexa-Valadares school (of the province of Pontevedra).

A meeting was then arranged with the parents/legal guardians of the children in these schools to explain the scope of the project, to answer any questions, doubts or any concerns about the project and also to highlight the expected outcomes for both the schoolchildren and their families.

The confidentiality of all data was assured, as well as the participant’s anonymity throughout the study process.

An informed consent document was provided, as well as a report summary of the information provided during the meeting. A period of 72 h was determined as an appropriate timeframe for the parents and guardians to consider the proposal and pose any other question that they still had, either to the school’s head office or to the main researcher. After the set time limit, the families returned the signed informed consent document, either authorizing or denying their child’s participation in the study.

**Figure 1:**
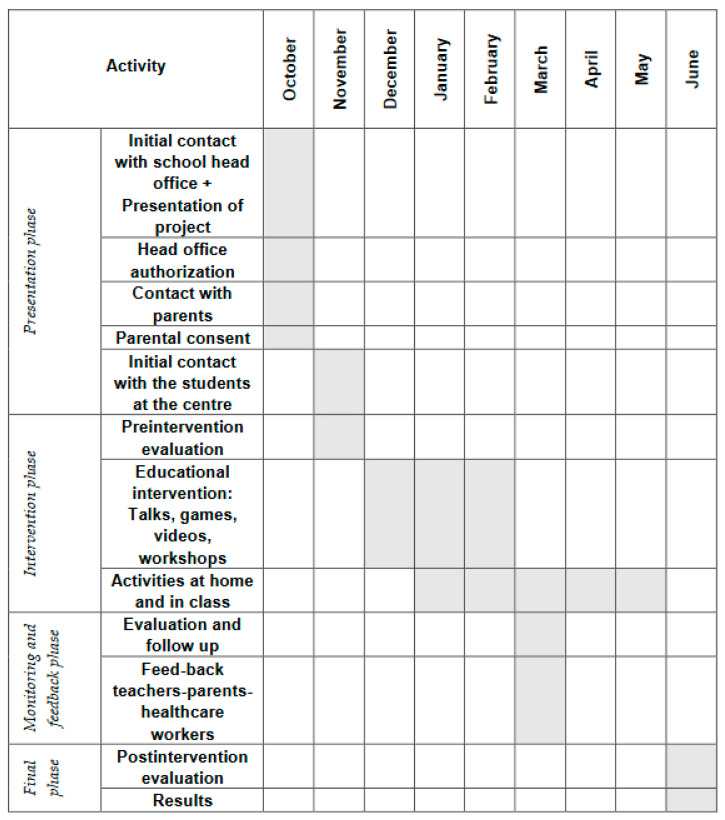
Intervention diagram.

The phases of the study are presented in the intervention diagram (Figure 1).

The diagram shows that there are different activities that are divided into stages.

Introductory phase: is the initial contact with the center, in which, the study was presented to the parents and/or guardians of the schoolchildren and their consent was requested for the development of the project.

Pre-intervention phase: is the first contact with the schoolchildren, where a properly adapted presentation of the project was made, as well as the first evaluation.

Intervention phase: this was performed through speeches on nutrition, video display, practical workshops and games on the topic, with the aim of training schoolchildren and parents in healthy habits and health education.

Progress and feedback phase: all parts were involved in this phase. Those that were head of the school, parents and those in charge of the research had a meeting to evaluate the progress of the study and consider possible improvements.

Final phase: the final evaluation was completed and the results were obtained.

All students who met the inclusion criteria of the study were invited to participate.

### 2.1. Inclusion Criteria

Students aged between 6 and 12 years from the three selected schools whose parents or legal guardians read and signed the informed consent document authorizing their participation in the study.

### 2.2. Exclusion Criteria

They did not respond freely and voluntarily to the survey.

Existing physical or psychological limitations that prevented the student from understanding the questions and answers of the questionnaire, or that prevented the child from participating in educational workshops, such as designing healthy menus.

### 2.3. Study Variables

We performed an initial evaluation by collecting data through an adapted questionnaire based on the enKid study pictograms [7]. The questionnaire enKid has 40 questions organized into themed blocks: basic diet; healthy diet; personal hygiene and health; healthy physical exercise and good posture habits; household chores and accident prevention (in our study, we only used the data related to diet).

Each block is preceded by an explicative statement that informs the student about what will be asked, and a list of good practices related to the thematic block at hand.

For the development of our study, we have focused on the questions on eating habits of the enKid questionnaire. Of the collected variables of the questionnaire on health habits, we considered the following:

Sociodemographic variables: age, sex (boy/girl), educational level of father and mother, place of residence (urban/rural), number of siblings.

Anthropometric variables: body mass index (BMI), where the weight (kg) was measured using a standardized scale ± 0.05 kg without shoes and wearing light clothing. Height was measured using a height rod ± 1 mm that is incorporated into the scale. Overweight and obesity are established when the body mass index (BMI = weight/height^2^ [kg/m^2^]) is above the respective cut off points, recommended by the International Obesity Task Force (IOTF) for each age group and sex of the child or adolescent. For the boys of our study, the IOTF defines them as overweight when the BMI is between the values 20.20 and 24.57; and as obese when the value of BMI is above 24.57. For girls of the same age, overweight is considered when the value of BMI is between 22.30 and 24.77 and obesity when the value is greater than 24.77. It is considered as low weight when the BMI is less than 18.5 [28].

### 2.4. Educational Intervention

We carried out the implementation of the educational intervention previously designed by establishing a series of fun and formative activities adapted to the different age groups of the children participating in the study focusing on healthy lifestyle habits.

The duration of the educational intervention was an academic year (28 weeks), with hour-long sessions twice a week, and it was based on motivating and implicating the students in the acquisition of healthy lifestyle habits by catching their attention with fun interactive activities, competitions and informative videos adapted to each age group.

Among the activities, we programmed didactic workshops with audio–visual resources, and we distributed a folder with games that would be carried out over the following months and a healthy eating pyramid magnet to stick on the refrigerator door. We also ran educational workshops for parents, legal guardians and teachers.

The intervention was designed and presented as a participative game both in the school setting and at home; in this way, we aimed for an active involvement of not only the children but also their families.

The activities took place outside of lesson time after the last morning period and before lunch break or were integrated into lessons of the subject Environment Sciences, following the schools’ recommendations. The programmed events were never organized as extracurricular activities in order to avoid work overload.

Later, in a more individualized manner, we programmed activities at the weekends with the collaboration of parents and guardians, such as designing healthy weekend meal plans, always taking into consideration each family setting. In these activities, each child participated with at least one of their parents or guardians.

Throughout the duration of the intervention, we monitored progress closely to verify the involvement of the students and their families. We obtained weekly reports from parents or guardians that reflected changes in attitudes observed in the children regarding the analyzed areas and gathering relevant queries or comments from the children.

We performed a final evaluation at the end of the seven months since intervention initiation and measured effectiveness of the educational intervention by using the same scales as those used in the initial evaluation.

### 2.5. Sample Size

We selected a sample of 479 students, an equivalent of 2% of all children aged 6 to 12 registered in the public schools of the provinces of Orense and Pontevedra during the academic school year 2015/2016.

The needed sample size of *n* = 479 students was calculated by establishing a confidence level of 95% and a margin of error of 5%, considering that the total number of students aged 6–12 registered in the public schools of the provinces of Ourense and Pontevedra was 24,900 students and supposing that, after the educational intervention, in the worst scenario, only 50% of the students would show improvements.

### 2.6. Statistical Analysis

We performed the Kolmogorov–Smirnov test to confirm the normal distribution of the variables. All of the variables complied with normality criteria, except for the type of food eaten for breakfast, lunch and supper. These variables were studied using non-parametric tests. We performed a descriptive analysis of the characteristics of the studied population. As inferential analysis, Student’s *t*-test was performed to compare the parametric variables, and the Mann–Whitney U test was applied to the non-parametric variables.

The quantitative variables were calculated using measures of central tendency (mean and median), dispersion (standard deviation) and position (limits of the distribution). The qualitative variables were expressed using absolute frequencies and relative frequencies (percentages). We set an α error of 5% (*p* ≤ 0.05). Statistical analysis was performed using the program IBM SPSS version 22.

## 3. Results

### 3.1. Description of the Participants

Finally, the sample size was 479 students registered during the 2014/2015 academic year.

**Figure 2:**
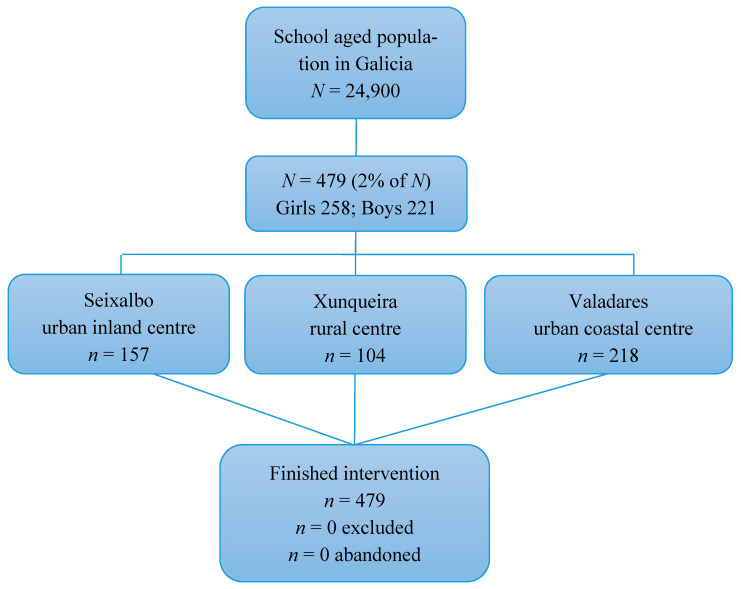
Flow diagram of the study sample.

The following figure shows a global description of our study sample (Figure 2).

Of the 479 children included in the study, 258 were girls and 221 were boys. The mean age of our sample was 8.7 (±1.5) years. Regarding participant population distribution according to geographic location, most of the children (322 (76.2%)) lived in an urban setting, and the school with the most participants was CEIP Igrexa-Valadares of Pontevedra.

Most of the students, 93.3%, had one sibling and only 4.2% had two siblings. Most of them lived with both parents, followed by parents and grandparents. Regarding the education level of the parents, most of the students had parents who had completed 6 years of secondary school (31.9%) or had pursued further studies (22.1%). With regards to parent occupation, 71.4% (342 children) were from homes where both parents worked and, in 135 families, the only breadwinner was the father (28.2%), (Table 1).

We found no significant relation between the family structure and BMI (*p* = 0.226), although the greatest percentage of children with low weight were from single-parent families or lived with their grandparents, and the greatest percentage of children who were overweight or obese lived with both parents and grandparents.

### 3.2. Eating Habits and Relation to BMI Pre-Intervention

Generally, 72% of the children had a BMI within normal values, and 24.2% of the sample population showed obesity (27.6% of the boys and 21.3% of the girls).

When considering the completion of five daily meals, 7 children (1.5%) had less than three meals a day, whereas 435 (90.8%) normally ate five meals a day. The most frequent meal was lunch (99.8%) and the least frequent was the afternoon snack (97.1%).

Regarding the foods eaten at each meal, 363 (75.8%) children drank milk at breakfast, most frequently accompanied with sugary treats (in 155 students). For their snack during mid-morning breaks, 279 (58.2%) children preferred sandwiches. For lunch, pasta was consumed by 139 (29%) children and 192 (40.1%) children ate different beans and pulses with white meats. White meats were also preferred for the evening supper. A total of 195 (40.7%) students reported eating sandwiches as their afternoon snack, many of them with fruit juice.

**Figure 3:**
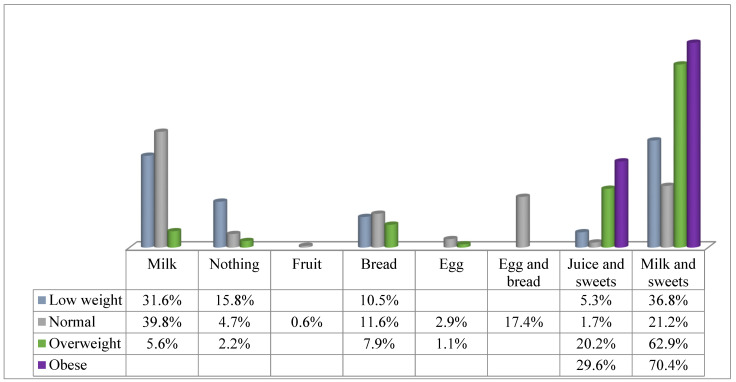
Food consumption at breakfast according to the BMI prior to the intervention.

The food consumption that the students made at breakfast according to the BMI before starting the intervention is reflected in Figure 3.

We found a significant relation between the type of food eaten at breakfast and BMI (*p* < 0.001).

We also found a statistically significant relation between BMI values and the types of food eaten for lunch (*p* < 0.001), where pasta was the most frequent food eaten by children who were overweight (32.5%) or obese (55.3%), whereas the combination of different beans and pulses and meat (52.7%) and sandwiches (5.3%) were most frequently eaten by children with low weight. The foods most frequently eaten by children with BMI values within the normal range were beans and pulses with meat (52.5%).

Regarding healthy foods, 379 (79.1%) children ate fruit daily, whereas only 129 (26.9%) ate vegetables on a daily basis. We found no significant relation between BMI values and the consumption of fruits, vegetables, sweets, pastas and meats. In addition, 82.3% ate fish more than twice a week.

### 3.3. Eating Habits and Relation to BMI Post-Intervention

After the intervention, we found a significant reduction in the children’s BMI (*p* < 0.001). Of the 89 overweight students, only 35 (39.3%) remained overweight, and of the 27 obese students, 26 (96.3%) lost weight so that 25 of them entered the overweight category and one reached BMI values within the normal range. None of the students with low weight increased their BMI (Table 2).

### 3.4. Study of the Variables Associated

The results of the educational intervention in healthy habits indicating the variables that improve and the beneficiaries in which they are statistically significant are shown in Table 3.

When analyzing the food consumption during different meals, we observed that those students who previously did not eat anything for breakfast started having mainly milk, juice or fruit. Of those who ate sugary snacks with milk or juice, many changed their diet by introducing bread and fruit to their breakfasts.

**Figure 4:**
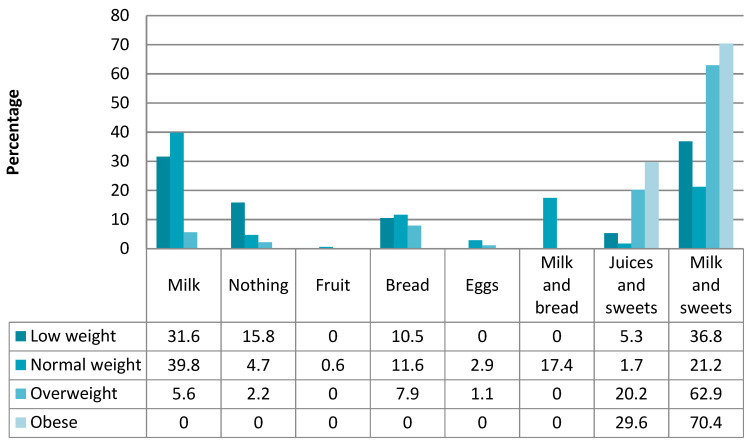
Food consumption at breakfast according to BMI before the intervention.

**Figure 5:**
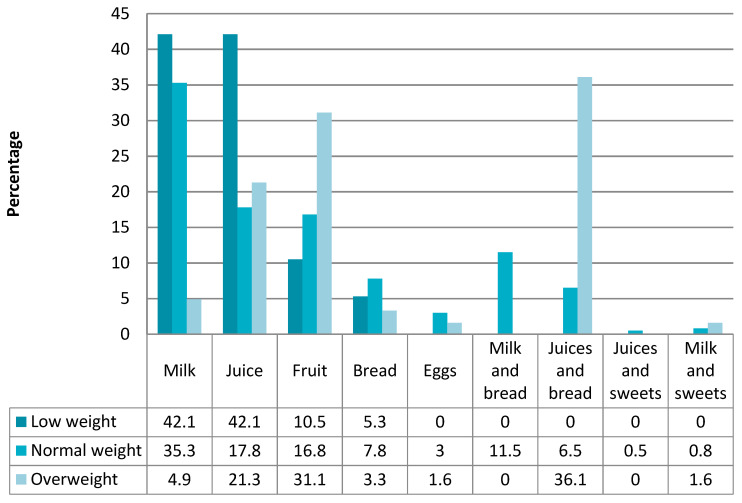
Food consumption at breakfast according to BMI after the intervention.

We found that the students with low weight tended to eat a single food for breakfast (milk, bread or juice), whereas most of the children who *were* overweight or obese ate juice and bread. (Figure 4 and Figure 5).

Another significant change (*p* < 0.001) was observed in the eating habits during the mid-morning break in the children who ate sugary *snacks*, either with or without dairy products, as they reduced their consumption of such foods and began eating mainly fruit and sandwiches. Focusing on sandwiches as mid-morning snacks, an increase of 7.1% was observed. Food consumption during the mid-morning break was also associated with BMI values (*p* < 0.001), where children with low weight ate the most dairy products (26.3%) and fruit (15.8%) and ate sandwiches the least (42.1%), compared to 64.4% in those with normal BMI values and 78.7% in those overweight.

We observed a reduction in the number of children who frequently (more than three times per week) ate pasta, with or without some form of meat, for lunch, from 139 to 19 students. These changes were also statistically significant (*p* < 0.001). Additionally, prior to the intervention, 38.6% ate pasta daily, a habit that was no longer observed after the intervention. The consumption of red and white meats combined with different beans and pulses nearly tripled, shifting from 132 to 331 children (69.1%) who frequently ate these foods, although two children maintained the habit of eating sandwiches for lunch (both with normal BMI values).

After the intervention, we found that the students with a normal BMI ate more pasta on a regular basis than those who were overweight or obese (*p* = 0.005), whereas, in four of the six children who ate eggs daily, their weight was above the normal weight range.

Generally, eating habits changed regarding the afternoon snack and evening supper. We observed an increase in the consumption of dairy products (from 14.6% to 35.6%), juices (from 2.9% to 6.7%) and fruits (from 19% to 34%) as the only food eaten for an afternoon snack. This implied a reduction in the consumption of sandwiches from 40.7% to 1.4% (*p* < 0.001). The consumption of sandwiches for supper also decreased (from 2.9% to 0.6%) and we observed an increase in the consumption of beans and pulses (11.7% to 23%).

## 4. Discussion

This research reinforces the role that schools play as a strategic sector in the improvement of health-related behaviors. In addition, the educational intervention includes several age groups considering children’s cognitive and social development.

There is a lower prevalence of obesity in our study population (27.6% of boys and 21.3% of girls) compared to the data published in the epidemiological bulletin of Galicia [29] from 2013, where 34.7% of boys and 35.1% of girls were obese.

The data obtained in this study show that underweight schoolchildren are also slightly higher, at 4% (4.1% in boys and 3.9% in girls), than the data reported in the 2013 Xermola plan (3.5% and 3.1%, respectively) [13]. Differences are also found with respect to the area where schoolchildren live, as a study by the Consellería de Sanidade de Galicia reported that 34.5% of children in urban areas and 46% of those in rural areas were above normal weight ranges, whereas, in this study, only 11.5% of children living in a rural location and 34.9% of those in urban areas had values above normal. These differences in the location of living may be related to the outdoor activities of children in rural contexts; schoolchildren living in urban areas did not report regular outdoor activities.

In Galicia, the study published by Lois and Rial [30], which evaluates the diet and physical activity in the 4th year of primary-school-aged children from a semi-private urban school, presents values in the overweight group that are lower than the values found in this study (10.76% in boys and 11.77% in girls).

When the results obtained before and after the educational intervention are analyzed, it is shown that underweight was maintained in both boys and girls; however, normal BMI values increased significantly in both boys and girls as a result of a reduction in overweight and obesity in both sexes. The schoolchildren who showed a significant improvement were those from an urban location, both seaside and countryside, although this should not be interpreted as a negative result of the intervention in rural schoolchildren, as these children already had the lowest levels of obesity.

In the study published by Bustamante et al. [31], they report an association between the parental socio-economic level and habitat that shows that more sedentary lifestyles and higher-socio-economic-class families have a much higher prevalence of overweight and obesity (20.9% and 5.9%, respectively) compared to the data obtained from lower-class families, where values were 13.7% and 2.2%, respectively.

This research studied the variable food consumption in relation to BMI. The results obtained after the intervention show positive changes in the food consumption patterns of schoolchildren. There is a higher consumption of pasta and carbohydrate-rich foods in children with higher than normal BMI values, as shown in the study published by Fajardo et al. [32].

After the intervention, those students who initially had higher BMI values significantly reduced their consumption of pasta for lunch and this change, along with the introduction of other foods with a lower fat content, was the factor that contributed to a decrease in overweight/obesity rates from almost 25% to 13% [33]. Another change that is likely to have contributed to this decrease in overweight/obesity rates is the eating of five meals a day, where we found that 8% of our study population initially did not eat five meals a day and that this percentage was reduced to 0.6%. In the book on psychology and nutrition published by Cabello et al. [34], they report that 10% of schoolchildren went to school without breakfast and 4.1% reported not having eaten anything during the morning. These values are much higher than those obtained in this study.

Overall, all school children improved their eating habits, increasing their consumption of healthy foods, such as vegetables, meats and fruits. Although, in this study, 33 children who ate fish on a daily basis reduced their fish consumption to only one to three times a week, this can be considered a positive result, as it is probably a result of these children including previously unusual foods in their diet [35]. Following this line of research is the study by Alvirde-García et al. [26], where they observed that, after an educational intervention, there was a significant reduction in the intake of bread, fat and sugary foods, in order to increase the consumption of fruit and vegetables.

There is no doubt that an adequate and healthy diet is essential during all stages of life, but even more so during the stages of childhood growth and development. In this sense, this research provides further evidence that it is necessary to be aware of eating habits at these stages in order to understand their physiological and functional implications, and because it is at these stages that long-lasting eating habits are established, where many of these habits will be carried into adulthood. Overweight and obesity have been found to be more prevalent in children who eat sugary snacks and dairy products [36].

It is necessary to highlight the effect of school intervention programs that are not designed correctly, as it is recommended that they have a duration of more than one year and have a multidisciplinary involvement of schoolchildren, families and schools to achieve the most effectiveness [37].

In terms of future lines of research, it may be interesting to develop studies on patterns of toxic habits and the influence of the family environment.

As for the limitations of this research, the fact that this study was implemented in only one region of Spain is a limitation in terms of location, and it would be interesting to extend this study to other regions or to extend it to a country-wide study. Another limitation came from the time limit established in this study, where the duration was only one academic year without the possibility of extending the continuity of the research, since the centers where the research was carried out do not provide secondary education, so the schoolchildren continue their studies in other educational centers.

## 5. Conclusions

The educational intervention reduced the levels of overweight and obesity in schoolchildren and improved their eating habits. However, it would be more appropriate to implement the educational interventions in a long-term perspective (more than one year).

Schoolchildren generally modified their eating habits in a positive way, including healthy foods (vegetables, meat and fruit) in their diet and reducing the consumption of sweets, sugary snacks and pasta.

Adequate nutrition and the establishment of healthy lifestyle habits in school children can help to not only prevent immediate health problems and promote a healthy lifestyle, but can also reduce the risk of developing future chronic diseases.

## Figures and Tables

**Table 1:** Descriptive analysis of sample.

Variables		Frequency (N)	Percentage (%)
Sex	Male	221	46.1
	Female	258	53.9
Age	Mean (deviation)	8.66 (1.489)	
BMI	Low weight	19	4
	Normal	344	71.8
	Overweight	89	18.6
	Obese	27	5.6
Location	Urban coastal	157	32.8
	Urban inland	104	21.7
	Rural inland	218	45.5
Lives with	Parents	306	63.9
	Parents + grandparents	101	21.1
	Others	72	15
Parental education level	University	106	22.1
	6 years Secondary	153	31.9
	Primary or incomplete secondary	220	45.9
Parental occupation	Father works	135	28.2
	Mother works	2	0.4
	Both work	342	71.4
Meal completion	Completes 5 meals	435	90.8
	Does not eat breakfast	6	1.3
	Does not eat mid-morning snack	12	2.5
	Does not eat lunch	1	0.2
	Does not eat afternoon snack	14	2.9
	Does not eat supper	11	2.3
Number of siblings	Only child	60	12.5
	1 sibling	399	83.3
	2 siblings	20	4.2

**Table 2:** Comparative analysis of BMI values pre- and post-intervention. Number and percentage (%).

BMI Pre-Intervention	BMI Post-Intervention			
	Low Weight	Normal Weight	Overweight	Obese
Total	19 (4%)	399 (83.3%)	60 (12.5%)	1 (0.2%)
Low weight 19 (4%)	19 (100%)	0 (0.0%)	0 (0.0%)	0 (0.0%)
Normal weight 344 (71.8%)	0 (0.0%)	344 (100%)	0 (0.0%)	0 (0.0%)
Overweight 89 (18.6%)	0 (0.0%)	54 (60.7%)	35 (39.3%)	0 (0.0%)
Obese 27 (5.6%)	0 (0.0%)	1 (3.7%)	25 (92.6%)	1 (3.7%)

**Table 3:** Results of the intervention according to beneficiary variables and beneficiaries of the improvement.

Improvement	Associated Variable	Beneficiaries of the Improvement	*p*-Value
BMI	Type of center	Urban coastal (21.7%) and inland (31.7%)	<0.001
	Previous BMI	Overweight (60.7%) and obesity (96.3%)	<0.001
Eating habits mid-morning break	Type of center	Urban coastal (21.7%)	0.001
	Lives with	Other family structure (31.9%)	<0.001
Pasta consumption	Type of center	Rural inland (89.9%) and urban inland (85.6%)	<0.001
	Parental education level	University (93.4%) and primary (83.6%)	0.003
Vegetable consumption	Lives with	Parents (65%) and parents + grandparents (71.3%)	<0.001
	Parental education level	Primary/secondary (71.4%)	0.001
	Parental occupation	Only one parent works (75.2%)	<0.001
Sweets consumption	Parental education level	Primary/secondary (74.5%) and university (61.3%)	<0.001
	Parental occupation	Only one parent works (80.3%)	<0.001
Hygiene habits: bathing	Type of center	Rural inland (28.4%)	0.001
	Lives with	Others (58.3%)	<0.001
	Parental education level	6 years secondary (39.2%) and university (32.1%)	<0.001
	Parental occupation	Both (27.2%)	<0.001

## Data Availability

Data are available on request from the corresponding author.
